# High-Performance Work Practices and Employee Wellbeing—Does Health-Oriented Leadership Make a Difference?

**DOI:** 10.3389/fpsyg.2022.833028

**Published:** 2022-03-03

**Authors:** Sven Hauff, Annika Krick, Laura Klebe, Jörg Felfe

**Affiliations:** Department of Humanities and Social Sciences, Helmut Schmidt University, Hamburg, Germany

**Keywords:** high-performance work practices (HPWPs), employee wellbeing (EWB), health-oriented leadership, staff care, health, commitment, engagement

## Abstract

This paper sheds further light on the contextual boundaries in the relationship between high-performance work practices (HPWPs) and employee wellbeing. In particular, we analyze whether this relationship is moderated by health-oriented leadership behavior (i.e., staff care) which describes the extent to which leaders value, are aware of, and protect their followers’ health at work. Our analyses are based on employee data (*N* = 1,345) from Germany, covering two points in time. Findings show positive associations between HPWPs and happiness-related (i.e., engagement, commitment) and health-related (i.e., general health, physical health complaints, mental health complaints, strain) wellbeing outcomes. The positive relationship between HPWPs and employee wellbeing is weaker the more employees experience leadership behavior in terms of staff care. Thus, our results provide further evidence for a substitutive or compensatory effect between HRM and leadership.

## Introduction

In the past years we have observed an increasing interest in the relationships between high-performance work practices (HPWPs; Huselid, 1995; Appelbaum et al., 2000) and employee related outcomes including different aspects of employee wellbeing like job satisfaction, commitment, and health (Peccei and Van De Voorde, 2019). However, despite numerous studies that have analyzed the impact of HPWPs on employee wellbeing (e.g., Jiang et al., 2012b; Ogbonnaya et al., 2017; Zhang et al., 2018; Ogbonnaya and Messersmith, 2019), we still do not know whether HPWPs are beneficial or a threat for employee wellbeing. Indeed, the inconclusive results nurture the debate on mutual gains vs. conflicting outcomes: some researchers argue that HPWPs do not only serve the organization but also contribute to increase the wellbeing of employees (the mutual gains or optimistic perspective), while others assume that HPWPs have a negative impact on employee wellbeing, since HPWPs are associated with an intensification of work and a more systematic exploitation of employees (the conflicting outcomes or pessimistic perspective; Ogbonnaya and Messersmith, 2019; Peccei and Van De Voorde, 2019).

Taken a diplomatic stance one could argue that the answer to this debate is: it depends. First, it depends on the context. Multiple contingency factors like workforce characteristics and the demographic composition of employees or organizational characteristics such as sector/branch of industry, and even national institutions or cultural differences may influence the relationships between HPWPs and employee wellbeing (Peccei et al., 2013). Thus, the relationships might be positive in some situations and negative in others. Second, it depends on the outcome in question. Employee wellbeing is a multidimensional construct, thus HPWPs can be positively related to some indicators of wellbeing (e.g., job satisfaction) and at the same time be negatively related to other indicators of wellbeing (e.g., health; Grant et al., 2007; Guerci et al., 2022).

With this paper we shed further light on these issues by analyzing whether health-oriented leadership (HoL; Franke et al., 2014) influences the relationship between HPWPs and employee wellbeing. Leadership behavior represents a key contextual factor since both HRM and leadership are engaged in people management and are crucial for employee wellbeing (Guest, 2017; Leroy et al., 2018). Recent research has already started to analyze the simultaneous influence of HRM and leadership to better understand the unique and joint effects (e.g., Marescaux et al., 2018; Wang et al., 2019; Ehrnrooth et al., 2020; Hauff et al., 2020; Jo et al., 2020). However, to our best knowledge no study has focused on leadership behavior that explicitly addresses employee wellbeing. This represents a major research gap since leadership behavior can, for example, influence the perception of HRM or can help organizations to achieve different, even contradictory goals (Leroy et al., 2018). Therefore, health-oriented leadership behavior—which includes leaders’ follower-directed behavior in terms of staff care (i.e., the extent to which leaders value, are aware of, and protect their followers’ health at work), but also covers leaders’ self care (i.e., the extent to which they value, are aware of, and protect their own health; Franke et al., 2014)—might be a key for organizations to either increase the positive influence of HPWPs on employee wellbeing (following the mutual gains or optimistic perspective), or to mitigate potential negative effects (the conflicting outcomes or pessimistic perspective).

Besides analyzing the influence of HoL in the relationship between HPWPs and employee wellbeing, we also contribute to the literature by integrating a broad set of employee wellbeing indicators, including organizational commitment, engagement, and physical and mental health. The integration of multiple indicators acknowledges the multidimensional nature of employee wellbeing and the possibility of potential trade-offs (Grant et al., 2007; Guerci et al., 2022).

## Theoretical Background

### High-Performance Work Practices and Employee Wellbeing

HPWPs are usually conceptualized based on the ability, motivation, opportunity (AMO) framework (Jiang et al., 2012b). The guiding idea is that high employee performance (and subsequently also high organizational performance) can only be achieved if employees have the necessary abilities (knowledge, skills), are highly motivated, and also have the opportunities to use their skills and motivation (Appelbaum et al., 2000; Hauff et al., 2021). Hence firms should invest in HRM practices that foster employees’ skills, motivation, and opportunities. For example, to increase employees’ abilities, firms should apply extensive recruitment and selection, and should offer comprehensive training. Crucial HRM practices to increase motivation are performance management, compensation, and incentive practices. Finally, job design, teamwork, and involvement practices are key for employees’ opportunities (Lepak et al., 2006; Jiang et al., 2012a,b; Hauff et al., 2018).

According to the mutual gains or optimistic perspective, investments in HPWPs do not only help organizations by increasing employee performance, they also increase employee wellbeing and therewith create a win-win situation. Multiple theories underpin this line of reasoning (Peccei et al., 2013; Peccei and Van De Voorde, 2019). Most often researcher refer to social exchange theory (Blau, 1964) and the norm of reciprocity (Gouldner, 1960) to explain the positive effects of HPWPs on employee wellbeing. Indeed, investments in HPWPs can signal employees that an organization is committed to them and their wellbeing. Employees respond to these signals with positive attitudes and behavior, like high job satisfaction, engagement, and commitment. Researchers have also referred to other theories to hypothesize potential positive effects of HPWPs on employee wellbeing. The most important are the Job Demands-Resources (JD-R) model (Demerouti et al., 2001), Self-determination theory (Deci et al., 1989), and Conservation of Resources (COR) theory (Hobfoll, 1989). Based on these theories it can be argued, for example, that HPWPs increase employee wellbeing since they include job resources that help employees to achieve their work-related goals and also to reduce or buffer the effects of stressors.

In contrast, the conflicting outcomes or pessimistic perspective assumes negative relationships between HPWPs and employee wellbeing. This line of reasoning is primarily rooted in labor process theory (Thompson and Harley, 2007). Central to this perspective is the idea that organizations constantly need to reduce cost and to increase quality and productivity to be able to compete in international markets. HPWPs can be considered as a means to achieve these goals, while threatening employee wellbeing. For instance, extensive training activities may increase job complexity, and performance management and compensation practices encourage high efforts. Further, autonomy, teamwork, and organizational participation could provide employees with additional responsibilities that require more efforts from them (Wang et al., 2019). Thus, while HPWPs are positive for organizational performance, they may lead to intensification, stress, and strain with detrimental effects on employee wellbeing (Peccei et al., 2013).

In a recent review, Peccei and Van De Voorde (2019) showed that HRM research provides more support for the mutual gains or optimistic perspective. However, research that analyses the influences of HPWPs on employee wellbeing partially shows a mixed picture, in particular when considering multiple dimensions of employee wellbeing. For instance, Ogbonnaya and Messersmith (2019) found that HPWPs increase affective commitment, but also job demands and stress. Similar, Guerci et al. (2022) found that most HPWPs are positively related to different dimension of employee wellbeing, but they also encountered trade-offs where HPWPs were positively related to some employee wellbeing dimensions, but negative with others.

Acknowledging potential demanding aspects of HPWPs and tradeoffs regarding different dimension of employee wellbeing, and in line with the general tendency in HRM research (Peccei and Van De Voorde, 2019), we expect positive relationships between HPWPs and employee wellbeing. Accordingly, we posit the following:

*Hypothesis 1: HPWPs are positively related to employee wellbeing.*

### Health-Oriented Leadership and Employee Wellbeing

Effective leadership should focus on ensuring the success of the organization which is not only measured by employees’ performance, but also by their health as a decisive success factor. Therefore, domain-specific leadership concepts that address employee health and wellbeing more specifically than general leadership concepts came to the fore in leadership research (Franke et al., 2014). Health-oriented leadership behaviors explain additional variance beyond transformational or general positive leadership behaviors, and thus specifically contribute to employees’ wellbeing beyond traditional leadership styles (Franke et al., 2014; Vincent-Höper and Stein, 2019; Kaluza et al., 2021). The health-oriented leadership concept (HoL) by Franke et al. (2014) includes employee-directed health-promoting leadership (i.e., leaders’ staff care) and health-promoting self-leadership (i.e., leaders’ and followers’ self care). Both components foster employee health and encompass three sub facets: (1) value (i.e., health-oriented attitudes and priority of health), (2) awareness (i.e., perception of overload, recognition of health-specific warning signals), and (3) behavior (i.e., specific health-enhancing action patterns and avoidance of health-endangering actions; Franke et al., 2014).

Previous studies support the validity of the concept and show empirical evidence for the effectiveness of HoL for employee health and wellbeing (Franke et al., 2014; Pundt and Felfe, 2017; Klug et al., 2019; Santa Maria et al., 2019; Arnold and Rigotti, 2020). In line with assumptions of the HoL concept, previous studies showed that staff care represents an external resource which is positively related to employees’ affective organizational commitment, job satisfaction, and performance, as well as negatively to employees’ depression, burnout, health complaints, and strain (Franke et al., 2014; Horstmann and Remdisch, 2016; Pundt and Felfe, 2017; Horstmann, 2018; Klug et al., 2019; Santa Maria et al., 2019; Vonderlin et al., 2020; Klebe et al., 2021a,b). Besides direct effects of staff care on employee health, staff care also indirectly affects employee health by encouraging employees to care for themselves in terms of self care (Franke et al., 2014; Horstmann, 2018; Santa Maria et al., 2019; Klebe et al., 2021b). Self care itself reflects an internal resource and increases health and wellbeing, while reducing presenteeism, work-family conflicts, strain, exhaustion, and health complaints (Franke et al., 2014; Pundt and Felfe, 2017; Santa Maria et al., 2019; Klebe et al., 2021b).

In accordance with these findings, we propose the following:

*Hypothesis 2: HoL in terms of staff care is positively related to employee wellbeing.*

### Interaction Between High-Performance Work Practices and Health-Oriented Leadership

While research on HRM and leadership has usually been separated, recently we find an increasing interest in the analysis of the unique and joint effects of HRM and leadership (e.g., Marescaux et al., 2018; Wang et al., 2019; Ehrnrooth et al., 2020; Hauff et al., 2020; Jo et al., 2020). HRM and leadership can either have independent effects or interact in the management of people (Leroy et al., 2018). *Independence* implies that HRM and leadership operate in separation from each other. Thus, even when they aim to achieve the same goal, there won’t be any reciprocal influence. This seems unlikely since leaders usually implement HRM practices (Guest and Bos-Nehles, 2013) and employees won’t be able to always separate between leadership behavior and HRM initiatives in their perception of the organization (Leroy et al., 2018). Regarding the interaction between HRM and leadership, two main perspectives can be distinguished. On the one hand, a *positive, mutually reinforcing* effect between HRM and leadership has been recognized (Jo et al., 2020). The general idea here is that HRM and leadership can increase each other’s effectiveness in a synergistic way if they are aligned and therewith send similar signals to employees which then represents a “powerful connection” between HRM and leadership (McClean and Collins, 2019). On the other hand, researchers have also postulated a *negative, substitutive effect* (Chuang et al., 2016; Ehrnrooth et al., 2020). This perspective is based on the substitutes-for-leadership theory (Kerr, 1977; Kerr and Jermier, 1978) according to which certain organizational conditions may substitute for or even neutralize the effects of leader behaviors. Turning the argument around, leader behavior can also be considered a substitute for HRM (Jiang et al., 2015; Chuang et al., 2016). The empirical evidence regarding this issue is mixed. For example, Ehrnrooth et al. (2020) and Jo et al. (2020) found support for the positive, mutually reinforcing effect, while Jiang et al. (2015), Chuang et al. (2016), and Hauff et al. (2020) found support for the negative, substitutive effect.

This ambiguity also applies to the interaction between HPWPs and HoL when influencing employee wellbeing. On the one hand, we can propose that HoL should reinforce the positive relationship between HPWPs and employee wellbeing. This proposition can be built on several arguments. First, it can be argued that the positive relationship between HPWPs and employee wellbeing will be reinforced through HoL since the alignment of both sends an even stronger signal to employees that their organization cares for them. Second, building on the JD-R model (Bakker and Demerouti, 2007), it can be argued that HoL represents a resource that helps employees to deal with potential demanding aspects of HPWPs which strengthens the positive relationship between HPWPs and employee wellbeing. Third, HoL can represent a perceptual filter on how employees see HPWPs (Leroy et al., 2018). Leaders’ attitudes and behaviors can influence how employees interpret and evaluate HPWPs (Nishii and Paluch, 2018; Wang et al., 2019), and high levels of staff care should leave employees with an optimistic bias regarding the intention of HPWPs.

On the other hand, there are plausible arguments why the positive relationship between HPWPs and employee wellbeing is weaker with high HoL. First, HoL may partly substitute or compensate the positive effect of HPWPs. HPWPs can improve employee wellbeing, particularly by providing a more interesting and more stimulating work environment (e.g., via autonomy and organizational participation) which leads to increased job satisfaction, engagement, and health (Hauff et al., 2020). Importantly, HPWPs can signal employees that their organization cares about their wellbeing. However, if leaders show behavior in terms of staff care, the potential value of HPWPs for employee wellbeing might be reduced. Staff care includes the provision of healthy working conditions and the information about health and safety issues. Furthermore, leaders who engage in staff care encourage employees to engage in healthy working behavior (Franke et al., 2014). Such a direct interaction between leaders and employees can become a more proximal source of employee wellbeing compared to more formal practices included in HPWPs which then become redundant. Second, as both HPWPs and HoL elevate the level of employee wellbeing, a ceiling effect may come into play. On a higher level of employee wellbeing there is less room for improvement than on lower levels. This may also weaken the relationship. In light of these rationales, we propose the following hypothesis:

*Hypothesis 3: HPWPs and HoL in terms of staff care interact with each other so that the positive relationship between HPWPs and employee wellbeing is weaker with higher staff care.*

## Materials and Methods

### Sample and Procedure

Data for the present study were collected using an online questionnaire. The online survey took place at two independent measurement points 2 months apart (t1: end of March 2021 up to mid-April 2021; t2: from mid-June up to July 2021). Participants were recruited via a market research company. A total of *N* = 1,345 employees completed both surveys of which 45% were male. Participants had a mean age of *M* = 44.63 (*SD* = 13.03) working in a wide range of branches (e.g., 18.5% public administration; 4.4% = nursing, medicine, and health; 11.1% = metal and electrical industry; 10.2% = banking and insurance; 4.6% = logistics, transport, and traffic; 8.6% = IT, telecommunication; 6.5% = trade; 6.5% = education and research). 32.8% reported to work in the public sector, while the majority (67.2%) reported to work in the private sector. One third of the employees had an academic degree (32.5%). About half of the employees (50.1%) reported to work in large companies with more than 500 employees, whereas 24.1% reported to work in companies between 100 and 500 employees and 25.8% in companies with less than 100 employees.

To analyze direct and interaction effects of HPWPs and staff care on outcomes, and to reduce the risk for common method bias (Podsakoff et al., 2013), HPWPs and staff care were assessed at t1 and outcomes 2 months later at t2.

### Measures

#### High-Performance Work Practices

In this study we follow Jiang et al. (2012b) to conceptualize HPWPs. Accordingly, we considered comprehensive selection and extensive investments in training as ability-enhancing HPWPs. We referred to formal appraisals, high salary, pay for performance, extensive benefits, career advancement prospects, and job security as motivation-enhancing HPWPs. We included autonomy, organizational participation, teamwork, formal grievance procedures, and information sharing as opportunity-enhancing HPWPs. Each of these practices were assessed by statements such as “My organization pays me a salary (including bonuses) that is above the industry average” or “My organization offers me monetary rewards for great effort and good performance (success bonuses, profit sharing, etc.).” In line with extant research (Hauff, 2021), we summarized all 13 HPWPs into an additive index. Cronbach’s Alpha was α = 0.91.

#### Health-Oriented Leadership

To assess health-oriented leadership, the staff care scale of the HoL instrument by Pundt and Felfe (2017) was used. Previous studies showed the validity of the HoL construct, its scales, and its sub dimensions (Franke et al., 2014; Santa Maria et al., 2019; Kaluza et al., 2021). Staff care was measured with 19 items, for example “My supervisor immediately notices when something is wrong with my health,” “My health is very important to my supervisor” or “My supervisor tries to reduce my work demands by optimizing my work routines (e.g., set priorities, ensure for undisturbed work, daily planning).” Cronbach’s Alpha was α = 0.94.

#### Physical and Mental Health Complaints

Health complaints were assessed by measuring common work relevant physical and somatic symptoms adapted from a scale developed by Mohr (1986). Participants were asked to rate the frequency they experienced each physical (5 items, “headache, back, shoulder or neck pain,” “eye problems,” “sleep disturbance,” “cardiopulmonary problems, hypertension,” “gastrointestinal problems”) and mental health complaints (3 items, “symptoms of depression and anxiety,” “exhaustion,” “social withdrawal”) within the past 4 weeks. For each scale a sum index was computed. The index for physical health complaints ranged from 5 to 25, the index for mental health complaints from 3 to 15. Cronbach’s Alpha was α = 0.80 for physical health complaints and α = 0.84 for mental health complaints.

#### Strain

Strain was assessed using the irritation scale by Mohr et al. (2005). Irritation encompasses symptoms of cognitive and emotional work-related strain. Due to parsimony, the scale was shortened to four items. Sample items were: “I have difficulty relaxing after work” and “I get irritated easily, although I don’t want this to happen.” Cronbach’s Alpha was α = 0.87.

#### General Health

To measure general health, we used a single-item measure taken from the German version of the Copenhagen Psychosocial Questionnaire (COPSOQ) which was shown to be reliable and valid (Nübling et al., 2006). Participants rated their general state of health on a scale from 0 = *worst conceivable health* to 10 = *best conceivable health*.

#### Commitment

Commitment was assessed by the COMMIT questionnaire (Felfe and Franke, 2012). The COMMIT is a psychometrically validated measure to assess commitment with different foci such as organization, form of deployment, team, supervisor, and occupation. Commitment encompasses three subscales: affective, normative, and calculative commitment. In the present study, we focused on affective organizational commitment. Due to parsimony, we shortened the original scale of five items to four items. Sample items were: “I feel a strong sense of “belonging” to my organization” or “I’m proud to be part of this organization.” Cronbach’s Alpha was α = 0.94.

#### Engagement

Work engagement was assessed using the ultra-short measure for work engagement (UWES-3) from Schaufeli et al. (2017) which is a reliable and valid indicator of work engagement. Sample items were: “Within the past 4 weeks, I have been full of exuberant energy at my work” or “Within the past 4 weeks, I have been completely absorbed in my work.” Cronbach’s Alpha was α = 0.91.

HPWPs, staff care, and commitment were rated on a five-point Likert-scale from 1 = *not at all true* to 5 = *completely true.* Strain, physical and mental health complaints, and engagement were rated on a five-point Likert-scale from 1 = *never* to 5 = *almost always.*

### Analytical Strategy

To test our hypotheses, we conducted a moderation analysis (model 1) using the SPSS macro PROCESS. Moderation analysis is a key approach to analyze the influence of contextual boundaries since it helps to understand if and how the relations between variables change depending on the values of other variables (Hayes, 2018). Accordingly, moderation analysis is a standard tool to assess whether the influence of HPWPs depends on specific circumstances (Jiang and Messersmith, 2018); and has been applied in previous studies that have addressed the interrelationships between HRM and leadership behavior (Ehrnrooth et al., 2020; Hauff et al., 2020). Staff care at t1 was modeled as the moderating variable modifying the relationship between HPWPs at t1 (independent variable) and the dependent variables general health, physical health complaints, mental health complaints, strain, engagement, and commitment at t2. To account for individual differences, we considered gender, age, and education as potential control variables, since these sociodemographic characteristics have been found to be connected to employee wellbeing (Appelbaum et al., 2000; Warr, 2007). Furthermore, we control for organization size as an organizational factor that can affect employee wellbeing (Kalleberg et al., 2009). Before computing the product of HPWPs and staff care, both variables were mean centered.

## Results

### Descriptive Analyses

HPWPs at t1 were positively related to general health (*r* = 0.25, *p* < 0.01), engagement (*r* = 0.48, *p* < 0.01), and commitment at t2 (*r* = 0.55, *p* < 0.01), and negatively related to physical and mental health complaints (*r* = –0.22, *p* < 0.01; *r* = –0.23, *p* < 0.01) and strain at t2 (*r* = –0.24, *p* < 0.01). Staff care at t1 also showed positive relationships with general health (*r* = 0.25, *p* < 0.01), engagement (*r* = 0.42, *p* < 0.01), and commitment at t2 (*r* = 0.50, *p* < 0.01), and negative relationships with physical and mental health complaints (*r* = –0.28, *p* < 0.01; *r* = –0.30, *p* < 0.01) as well as strain at t2 (*r* = –0.29, *p* < 0.01). Means, standard deviations, and intercorrelations for the study variables are presented in Table 1.

**TABLE 1:** Descriptives and correlations of the study variables.

No.	Variable	*M*	*SD*	1	2	3	4	5	6	7	8	9	10	11	12
1	Gender	–	–	–											
2	Age	44.63	13.03	–0.04	–										
3	Education	4.83	1.12	−0.09**	0.04	–									
4	Organizational size	4.00	1.28	−0.10**	0.02	0.09**	–								
5	HPWPs t1	41.07	10.96	−0.13**	–0.03	0.08**	0.12**	(0.91)							
6	Staff care t1	2.65	0.81	−0.12**	−0.09**	–0.04	0.01	0.60**	(0.94)						
7	General health t2	7.54	2.04	−0.12**	−0.09**	0.05*	–0.01	0.29**	0.25**	–					
8	PHC t2	11.90	4.30	0.28**	−0.07*	−0.10**	–0.04	−0.22**	−0.28**	−0.58**	(0.80)				
9	MHC t2	7.19	3.21	0.24**	−0.07**	−0.05*	–0.05	−0.23**	−0.30**	−0.60**	0.78**	(0.84)			
10	Strain t2	2.42	0.90	0.14**	−0.09**	–0.01	–0.03	−0.24**	−0.29**	−0.44**	0.62**	0.67**	(0.87)		
11	Commitment t2	3.27	1.04	−0.07*	0.04	–0.02	0.03	0.55**	0.50**	0.28**	−0.27**	−0.33**	−0.29**	(0.94)	
12	Engagement t2	2.86	0.93	–0.05	0.03	–0.04	–0.05	0.48**	0.42**	0.37**	−0.36**	−0.41**	−0.36**	0.64**	(0.91)

*N = 1,345–2.188 due to listwise deletion, PHC, Physical health complaints; MHC, Mental health complaints. Cronbach’s Alpha in parentheses across the diagonals;*

* p < 0.05; ** p < 0.01.

### Testing Hypotheses

In H1 we postulated a positive relationship between HPWPs and indicators of employee wellbeing. The regression analyses showed positive main effects of HPWPs on general health [*B* = 0.03, *SE* = 0.01, *t* = 4.73, *p* < 0.001, 95% CI (0.02, 0.04)], engagement [*B* = 0.03, *SE* = 0.00, *t* = 11.73, *p* < 0.001, 95% CI (0.03, 0.04)], and commitment [*B* = 0.04, *SE* = 0.00, *t* = 15.22, *p* < 0.001, 95% CI (0.04, 0.05)]. Negative main effects of HPWPs were found for physical health complaints [*B* = –0.06, *SE* = 0.01, *t* = –4.81, *p* < 0.001, 95% CI (–0.09, –0.04)], mental health complaints [*B* = –0.04, *SE* = 0.01, *t* = –3.81, *p* < 0.001, 95% CI (–0.06, –0.02)], and strain [*B* = –0.01, *SE* = 0.00, *t* = –4.73, *p* < 0.001, 95% CI (–0.02, –0.01)]. Employees working in organizations implementing HPWPs were healthier, had less physical and mental health complaints, were less strained, more engaged, and more committed. Therefore, H1 was supported.

In H2 we expected a positive relationship between staff care and indicators of employee wellbeing. Results showed positive main effects of staff care on general health [*B* = 0.35, *SE* = 0.08, *t* = 4.21, *p* < 0.001, 95% CI (0.19, 0.52)], engagement [*B* = 0.25, *SE* = 0.03, *t* = 7.19, *p* < 0.001, 95% CI (0.18, 0.32)], and commitment [*B* = 0.33, *SE* = 0.04, *t* = 9.20, *p* < 0.001, 95% CI (0.26, 0.40)]. Staff care showed negative main effects on physical health complaints [*B* = –0.84, *SE* = 0.17, *t* = –4.92, *p* < 0.001, 95% CI (–1.17, –0.50)], mental health complaints [*B* = –0.79, *SE* = 0.13, *t* = –6.13, *p* < 0.001, 95% CI (–1.04, –0.53)], and strain [*B* = –0.21, *SE* = 0.04, *t* = –5.74, *p* < 0.001, 95% CI (–0.28, –0.14)]. Employees whose leaders showed more staff care were generally, physically, and mentally healthier, experienced less strain, were more engaged, and showed higher commitment to the organization. H2 was supported.

**FIGURE 1:**
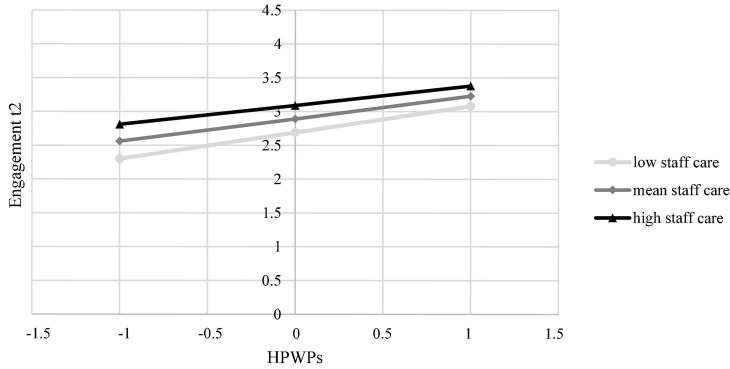
The conditional effect of HPWPs on employees’ engagement at t2 probed at –1SD, mean, and + 1SD for staff care.

**FIGURE 2:**
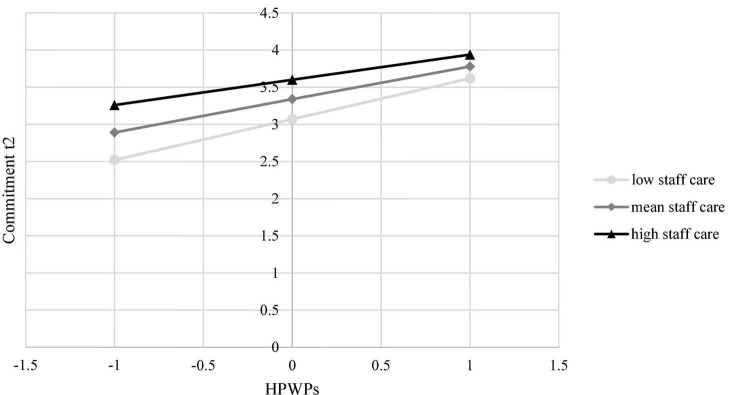
The conditional effect of HPWPs on employees’ commitment at t2 probed at –1SD, mean, and + 1SD for staff care.

In H3 we postulated that HoL in terms of staff care reduces the relationship between HPWPs and employee wellbeing. Overall, the moderation models accounted for significant variance in general health [*R*^2^ = 0.10, *F*(7, 1327) = 20.01, *p* < 0.001], physical and mental health complaints [*R*^2^ = 0.16, *F*(7, 1327] = 36.67, *p* < 0.001 and *R*^2^ = 0.15, *F*(7, 1327) = 32.27, *p* < 0.001, respectively], strain [*R*^2^ = 0.12, *F*(7, 1327) = 25.97, *p* < 0.001], engagement [*R*^2^ = 0.27, *F*(7, 1327) = 69.33, *p* < 0.001], and commitment [*R*^2^ = 0.38, *F*(7, 1327) = 116.75, *p* < 0.001]. However, only for engagement [*R*^2^ change = 0.004, *F*(1, 1327) = 6.95, *p* < 0.01] and commitment [*R*^2^ change = 0.013, *F*(1, 1327) = 28.55, *p* < 0.001], *R*^2^ significantly increased due to the interaction term. HPWPs had a conditional direct effect on employee engagement [*B* = –0.01, *SE* = 0.00, *t* = –2.64, *p* < 0.01, 95% CI (–0.01, –0.00)] and commitment [*B* = –0.01, *SE* = 0.00, *t* = –5.34, *p* < 0.001, 95% CI [–0.02, –0.01)] depending on staff care. Regarding engagement, this effect was coeff. = 0.04 [*SE* = 0.00, *t* = 11.30, *p* < 0.001, 95% CI (0.03, 0.04)] for low staff care and coeff. = 0.03 [*SE* = 0.00, *t* = 8.01, *p* < 0.001, 95% CI (0.02, 0.03)] for high staff care. Results showed that the simple slopes for both low and high staff care were significant (Figure 1). In case of high staff care, the effect of HPWPs on engagement was significantly reduced. Regarding commitment, this effect was coeff. = 0.05 [*SE* = 0.00, *t* = 15.78, *p* < 0.001, 95% CI (0.05, 0.06)] for low staff care and coeff. = 0.03 [*SE* = 0.00, *t* = 9.31, *p* < 0.001, 95% CI (0.03, 0.04)] for high staff care. Results showed that the simple slopes for both low and high staff care were significant (Figure 2). In case of high staff care, the effect of HPWPs on commitment was significantly reduced. There were no moderation effects of staff care on the relationships between HPWPs and general health, physical and mental health complaints, and strain. Therefore, H3 was partly supported for commitment and engagement. Regression coefficients, standard errors, confidence intervals, and explained variance for all moderation analyses are presented in Table 2.

**TABLE 2:** Moderation analyses to predict wellbeing.

	Wellbeing indicators at t2											
	
	General health		PHC		MHC		Strain		Engagement		Commitment	
						
Predictors at t1	Coeff.	SE	Coeff.	SE	Coeff.	SE	Coeff.	SE	Coeff.	SE	Coeff.	SE
Constant	8.22***	0.35	12.93***	0.71	7.75***	0.54	2.57***	0.15	3.18***	0.14	3.34***	0.15
HPWPs (H1)	0.03***	0.01	−0.06***	0.01	−0.04***	0.01	−0.01***	0.00	0.03***	0.00	0.04***	0.00
Staff care (H2)	0.35***	0.08	−0.84***	0.17	−0.79***	0.13	−0.21***	0.04	0.25***	0.03	0.33***	0.04
Staff care × HPWPs (H3)	0.00	0.01	0.01	0.01	0.01	0.01	–0.00	0.00	−0.01**	0.00	−0.01***	0.00
Age	−0.01*	0.00	−0.03**	0.01	−0.02**	0.01	−0.01***	0.00	0.01**	0.00	0.01***	0.00
Gender	−0.36**	0.11	1.98***	0.22	1.23***	0.17	0.17***	0.05	0.01	0.05	0.03	0.05
Education	0.08	0.05	−0.29**	0.10	–0.09	0.07	0.01	0.02	–0.03	0.02	–0.02	0.02
Organizational size	−0.10*	0.04	0.10	0.09	0.03	0.07	0.02	0.02	−0.10***	0.02	−0.07***	0.02
*R* ^2^	0.095***		0.162***		0.145***		0.121***		0.268***		0.381***	

Unstandardized regression coefficients, SE = standard error, PHC = Physical health complaints, MHC = Mental health complaints; * p < 0.05; ** p < 0.01; *** p < 0.001.

## Discussion

The aim of this paper was to deepen the understanding of the relationship between HPWPs and employee wellbeing. In line with the growing interest on the joint effects of HRM and leadership, we analyzed whether HoL in terms of staff care moderates the relationship between HPWPs and employee wellbeing. Thereby, we also considered multiple wellbeing outcomes which reflect different dimensions of employee wellbeing.

First, our findings provide further support for the mutual gains or optimistic perspective in HRM research according to which HPWPs do not only benefit the organization by increasing employee performance, but also employees themselves by increasing their wellbeing. Previous findings on the relationship between HPWPs and employee wellbeing have often been mixed. For example, Van De Voorde et al. (2012) found in their review that the studies that addressed the association between HPWPs and employee wellbeing in terms of happiness and social wellbeing mostly found a positive relationship (i.e., HPWPs usually increased happiness and social wellbeing), while the studies that addressed the association between HPWPs and employee health mostly reported a negative relationship (i.e., HPWPs mostly reduced health). In contrast to such mixed results (for more recent studies with mixed results see, e.g., Ogbonnaya and Messersmith, 2019), our findings showed positive relationships between HPWPs and all the considered wellbeing related outcomes. This is particularly noteworthy because we have taken into account various aspects of health-related wellbeing which has mostly been excluded or not been covered comprehensively in previous research (Peccei and Van De Voorde, 2019). Since our findings show a positive relationship not only with happiness-related wellbeing (i.e., engagement and commitment) but also with all the health-related wellbeing outcomes, they provide a rather strong support for the mutual gains or optimistic perspective. However, we do not want to get too optimistic and make bold prescriptions to practitioners, since we think it is still important to ask why research partially provides inconsistent results on the relationship between HPWPs and employee wellbeing. Potential reasons could be the level of analysis (we have only considered overall effects that reflect on the HRM system, but single HPWPs, subsets or different combination of HPWPs can have a differentiated effect; see, e.g., Guerci et al., 2022), the implementation and attribution of HPWPs (Nishii et al., 2008; Guest and Bos-Nehles, 2013), or the specific context (we have only analyzed the influence of leadership behavior, but other contingencies might be important; Peccei et al., 2013).

Second, we found that HoL in terms of staff care has positive relationships with the different indicators of employee wellbeing. This is in line with previous research which has shown evidence for positive associations between staff care and health indicators (Franke et al., 2014; Horstmann and Remdisch, 2016; Pundt and Felfe, 2017; Horstmann, 2018; Klug et al., 2019; Santa Maria et al., 2019; Vonderlin et al., 2020; Klebe et al., 2021a,b), and highlights the notion that staff care serves as an external resource for employees. Employees with leaders showing high levels of staff care are healthier (in terms of general health and less health complaints, less strained), more engaged, committed, and satisfied with their job.

Third, regarding the interaction between HPWPs and HoL, we have assumed that HoL in terms of staff care should weaken the relationship between HPWPs and employee wellbeing. This assumption was partially supported since we found that HPWPs had a conditional direct effect on employee engagement and commitment depending on staff care. The respective interaction terms were negative, thus our results provide further evidence for a substitutive or compensatory effect between HRM and leadership (Chuang et al., 2016; Ehrnrooth et al., 2020). Therewith, our results highlight that context matters: HPWPs can increase employee wellbeing, but their role for employee engagement and commitment is less important when employees perceive that their leaders show behavior in terms of staff care. This indicates that a direct exchange between leaders and employees can serve as a more proximal source of employee wellbeing in comparison to more formal HRM practices that are not explicitly designed to increase employee wellbeing. This interpretation also relates to the discussion on HRM practice salience according to which the influence of HRM practices depends on their visibility to employees (individually or collectively; Garg et al., 2021). Accordingly, the more leaders show behavior in terms of staff care, the more this behavior becomes a salient stimulus which stands out relative to HPWPs, and in turn reduces the influence of HPWPs. Nevertheless, again we would suggest not jumping to general conclusions about the nature of the relationship between HRM and leadership. Leroy et al. (2018) have argued that HRM and leadership can support each other leading to synergistic effects and several empirical studies have found evidence for such reinforcing mechanisms (e.g., Ehrnrooth et al., 2020; Jo et al., 2020). These mixed results might be related to the specific outcome in question, or might be triggered by other contextual factors (Chuang et al., 2016).

Regarding our health-related outcomes, we did not find any interaction between HPWPs and HoL. This could imply that HRM and leadership behavior can have independent effects on employee health. This interpretation should, however, also be viewed with some caution. Hauff et al. (2020) have shown that the relationship between HPWPs and employee health is mediated by job satisfaction and engagement. Thus, the conditional effect of HPWPs on engagement and commitment could spill over to employee health leading to an indirect conditional effect of HPWPs on employee health.

### Practical Implications

Our findings further support that it is worth to invest resources in the implementation of HPWPs and the development of HoL in order to foster employee wellbeing. HPWPs are supposed to increase employee performance by ensuring employees’ abilities (knowledge, skills), motivation, and opportunities to perform. Building on our findings we can say that HPWPs are not only a means to increase performance, but also to foster employee wellbeing. However, given the mixed results in previous research, we suggest that organizations who implement these practices should monitor the effects across time in order to be able to identify potential negative effects. Likewise, our findings also indicate the importance of HoL for employee wellbeing. Organizations who want to increase the wellbeing of their employees are therefore advised to evaluate their leaders’ behavior and to invest in specific trainings if they encounter deficits. When investing in HRM and leadership, organizations should be aware of the boundary conditions between those two: investments in HRM can increase employee wellbeing especially when employees do not experience staff care, but they will be less effective for employee wellbeing if employees have a strong perception that their supervisor cares about them and already supports their wellbeing.

### Limitations

Our findings should be cautiously interpreted, as this study is not without limitations. First, we used employee level data which only covers the subjective perceptions of these employees, but not objective measures. However, constructs like engagement, commitment, and health require introspection which makes individuals the most appropriate source of information (Chan, 2009). Similar, the subjective perception of HRM practices and leader behaviors should be more important for employees wellbeing compared to intended HRM strategies or self-perception of leaders (Schyns and Schilling, 2013; Han et al., 2020). Second, we have used a composite score to analyze the influence of HPWPs and did not capture differentiated effects and interactions among the single HPWPs (Hauff, 2021). This strategy allowed us to analyze the overall relationship between HPWPs and employee wellbeing as well as the interactions with HoL as a starting point for more detailed analyses.

### Future Research

There are several avenues for future research. First, as mentioned above, we think that research should further explore how the nature of the relationship between HPWPs and employee wellbeing is influenced by different levels of aggregation (i.e., individual practices, subsets of practices), the implementation and perception of HPWPs, as well as the specific context. Second, the interplay between HRM and leadership might be more complex than expected. From a temporal perspective, it might be possible that HRM practices might foster or hinder specific leadership behavior and vice versa. Furthermore, researcher should also consider that HRM practices are usually implemented across organizational units while leader behavior is team focused. Multi-level analyses might provide more detailed insights about the relationship between HRM and leadership at different levels of analysis including cross-level influences.

## Conclusion

We show that the relationship between HPWPs and employee wellbeing depends on the perceived level of HoL. In particular, our results indicate positive associations between HPWPs and happiness-related (i.e., engagement, commitment) and health-related (i.e., general health, physical health complaints, mental health complaints, strain) wellbeing outcomes, but these relationships are weaker the more employees experience leadership behavior in terms of staff care. Therewith, we shed further light on the contextual boundaries in the relationship between HPWPs and employee wellbeing and highlight that context indeed matters.

## Data Availability Statement

The datasets presented in this article are not readily available because of privacy restrictions. Requests to access the datasets should be directed to JF, felfe@hsu-hh.de.

## Author Contributions

SH and JF developed the research question and contributed to conception and design of the study. SH wrote the first draft of large parts of the manuscript. AK wrote a smaller part of the theoretical background, as well as “Materials and Methods and Results section,” closely supported by LK. JF and AK performed the statistical analysis. All authors involved in the development of the questionnaire and the organization of the database, contributed to manuscript revision, read, and approved the submitted version.

## Conflict of Interest

The authors declare that the research was conducted in the absence of any commercial or financial relationships that could be construed as a potential conflict of interest.

## Publisher’s Note

All claims expressed in this article are solely those of the authors and do not necessarily represent those of their affiliated organizations, or those of the publisher, the editors and the reviewers. Any product that may be evaluated in this article, or claim that may be made by its manufacturer, is not guaranteed or endorsed by the publisher.
